# Machine Learning Approaches to TCR Repertoire Analysis

**DOI:** 10.3389/fimmu.2022.858057

**Published:** 2022-07-15

**Authors:** Yotaro Katayama, Ryo Yokota, Taishin Akiyama, Tetsuya J. Kobayashi

**Affiliations:** ^1^ Graduate School of Engineering, The University of Tokyo, Tokyo, Japan; ^2^ National Research Institute of Police Science, Kashiwa, Chiba, Japan; ^3^ Laboratory for Immune Homeostasis, RIKEN Center for Integrative Medical Sciences, Yokohama, Japan; ^4^ Graduate School of Medical Life Science, Yokohama City University, Yokohama, Japan; ^5^ Institute of Industrial Science, The University of Tokyo, Tokyo, Japan

**Keywords:** machine learning, deep learning, T cell, T cell receptor, immunoinformatics

## Abstract

Sparked by the development of genome sequencing technology, the quantity and quality of data handled in immunological research have been changing dramatically. Various data and database platforms are now driving the rapid progress of machine learning for immunological data analysis. Of various topics in immunology, T cell receptor repertoire analysis is one of the most important targets of machine learning for assessing the state and abnormalities of immune systems. In this paper, we review recent repertoire analysis methods based on machine learning and deep learning and discuss their prospects.

## Introduction

Our bodies are constantly exposed to threats from various pathogenic bacteria, viruses, and cancer cells. The immune system is central to maintaining our body in a healthy state by detecting and evicting those pathogens. Among the different types of immune cells, T cells play various roles in the recognition, memory, and eviction of such threats (1). The peptides derived from those pathogens provide information to T cells when they are presented on the major histocompatibility complex (MHC) as antigens. T cells recognize an antigen if their T cell receptors (TCRs) can bind to the antigen-MHC complex. As antigens are diverse and MHC genes are highly polymorphic, TCRs also must be diverse to recognize a wide range of antigens. TCR diversity is generated by V(D)J recombination (2), one of the somatic recombination processes in our body. This process can potentially yield more than 10^13^ patterns of TCR (3). This diversity of TCRs ensures that, even if unknown antigens enter the body, there will be T cells with TCRs that can recognize them with a high probability. Furthermore, the recognition of such antigens by T cells, i.e., the binding of antigens to their TCRs, activates the T cells, inducing their proliferation and/or phenotypic changes (1). These dynamics alter the diversity of TCRs (TCR repertoire) in a T cell population and modulate its collective recognition of antigens. Therefore, quantitative evaluation of the TCR repertoire in individuals enables us to capture the individual’s past and present immunological status. It may also be possible to predict its future. Specifically, quantitative measurement of TCR repertoires may contribute to the quantification of abnormalities in the immune status of patients with specific diseases, the identification of the causes, and prediction of the risk of developing immune-related diseases in the future. For example, a diagnosis for a kind of leukemia is already approved by FDA (U.S. Food and Drug Administration) and clinically used. Quantitative measurement of TCR repertoire is performed by sequencing the recombinant genes encoding the TCRs of T cells in blood or other specimens. Since the mainstream of DNA sequencing technology has shifted from the low-throughput Sanger method to high-throughput next-generation sequencers (NGSs), the cost and time required for sequencing TCR repertoires have been dramatically reduced, which makes it practical to exploit TCR repertoires for practical applications. The recent advent of new techniques such as single-cell sequencing further provides ways to characterize different aspects of T cell repertoires (4).

In parallel with the development of TCR repertoire sequencing technology, bioinformatic and machine learning (ML) based data analysis, including deep learning (DL), is pervading the field of immunology. As we will see in more detail later, this is because a typical sample of repertoire data from a single person consists of a set of several hundred thousand sequences, and ML is an effective tool for extracting information from such a large amount of data. ML is already indispensable to repertoire sequencing analysis. It has also allowed new applications based on the repertoire sequencing such as the personal cancer vaccine (neoantigen vaccine) design (5) and the new testing methods for infections such as COVID-19 (6). Not only clinical applications, but also basic researches are assisted by ML based analysis methods. The impact of ML in repertoire sequencing is rapidly growing.

In this paper, we will outline the rapidly developing TCR repertoire analysis methods based on ML with useful tools and databases. We also discuss possible directions for further development of TCR repertoire analysis.

### Diversification of TCR

T cell progenitors are generated from hematopoietic stem cells in the bone marrow and undergo differentiation for maturation in the thymus before being exported to the periphery (1). A TCR is a heterodimer of *α*- and *β*-chains (certain TCRs consist of the *γ*- and *δ*-chain, but these are omitted here for simplicity). In the V(D)J recombination, one gene is selected from each of the V, D, and J gene groups of pre-recombinant genes of each chain (in *α*-chain, the D gene group does not exist), and the selected genes are combined with random insertions and deletions. Because of the randomness in the gene selection, insertions, and deletions, a variety of TCRs are generated. For example, in the case of the human *β*-chain, there are 64-67 V genes, 2 J genes, and 14 D genes according to IMGT database^1^. It should be noted that there are two loci for each TCR chain as humans are diploid. 30% of T cells (dual TCR) have two different productive TCR *α*-chain mRNAs despite the allelic exclusion mechanisms (7).

A TCR recognizes antigens present on the MHC. Antigens are digested into short peptides and presented on the MHC to form peptide-loaded MHC (pMHC) complexes (8). The affinity of a TCR to an pMHC complex is mainly determined by the recombination-dependent highly variable regions called the complementarity determining regions (CDRs) (9). In the sequence of recombinant TCR genes, three CDRs exist, from CDR1 to CDR3. CDR1 and CDR2 engage in binding to the MHC complex presenting an antigen, whereas CDR3 contributes to the binding affinity of the TCR to the antigen itself. Thus, the sequence of CDR3 plays a particularly important role in analyzing repertoires. Many studies to be introduced here also work on CDR3. After recombination, T cells in the thymus undergo positive and negative selection based on their interactions with self-antigens presented by other cells such as thymic epithelial cells (10). In positive selection, T cells with TCRs that have a moderate affinity to some self-antigen-MHC complexes are selected to survive. This process also selects T cells such that they recognize the antigen only if it is presented on the MHC (11). This phenomenon is called MHC restriction (12). Note that TCRs are “personalized” in this process as the MHC genes are highly polymorphic. The impact of genetic background, including MHC polymorphisms, on repertoire dynamics will be revisited in the next section. Cross-reactivity ensures that the selected T cells may recognize some non-self-antigen-MHC complexes too (13). In contrast, T cells with TCRs that have a high affinity to any self-antigen-MHC complex are eliminated in negative selection. This process decreases the number of self-reactive T cells by 60-70% (14). The remaining self-reactive T cells are suppressed by peripheral tolerance (14). By combining these mechanisms, TCRs that can recognize non-self-antigens but do not recognize self-antigens are selected and exported to the periphery. Then, T cells are induced to differentiate and proliferate depending on the antigens encountered in the periphery. From such peripheral T cell population dynamics, an appropriate repertoire is shaped and maintained so that it attains the ability to remember and rapidly respond to experienced antigens while retaining the diversity to respond to unknown ones (15).

### Influencing Factors of TCR Repertoire

Various factors affect the formation of a TCR repertoire. As we described earlier, peripheral antigen exposure changes the TCR repertoire. We review other potential factors in this section.

First, the genetic background can affect diverse aspects of repertoire dynamics. As we mentioned in the positive selection in the thymus, TCRs are selected to have MHC restriction. Therefore, the MHC type can influence the formation of repertoire. For example, associations between specific HLA (human MHC) types and specific sequences are observed (16). Furthermore, gene usage in V(D)J recombination might be affected by MHC (17). In addition, some HLA variants are associated with onset of autoimmune diseases (18). These results contrast with those of immunoglobulin for which V(D)J recombination process before selection is found to be highly different even between monozygotic (MZ) twins (19). Moreover, as HLA genes are highly variant (12), TCRs that bind to the same peptide can differ between people. Therefore, we cannot easily assume that T cells with the same TCR recognize the same antigens in different individuals when genetic information such as HLA is not the same.

Not only MHC, but also V(D)J genes themselves have polymorphisms (20, 21). Some of those variants are shown to affect the affinity of TCR-pMHC complex (22), which may result in different repertoire dynamics. We do not fully understand the effect of these variants on repertoire, as many variants remain to be discovered (23). Furthermore, genetic background is not the only dominant factor in the final peripheral repertoire dynamics. For example, a study in MZ twins revealed that peripheral repertoires of MZ twins are almost as different as those of unrelated individuals in terms of shared TCRs (24). On the other hand, those of the same person are very similar even after years (24). This might be caused by the fact that the probability of generation of the same TCR in different individuals is very low even if the MHC alleles are the same.

Second, aging also affect repertoire dynamics greatly. Age-related changes in the immune system are collectively called “immunosenescence” (25, 26). In the context of TCR repertoire analysis, immunosenescence often refers to the decrease in the proportion of naïve T cells and the increase in that of memory T cells undergoing persistent selection, for example, memory T cells recognizing antigens behind chronic viral infections such as cytomegalovirus (CMV) (27). This phenomenon impairs the diversity of the TCR repertoire. One of the main causes of this change is the decrease in the thymic output of naïve T cells due to the age-related thymic involution (28).

### Repertoire Sequencing and Batch Effects

**Figure 1:**
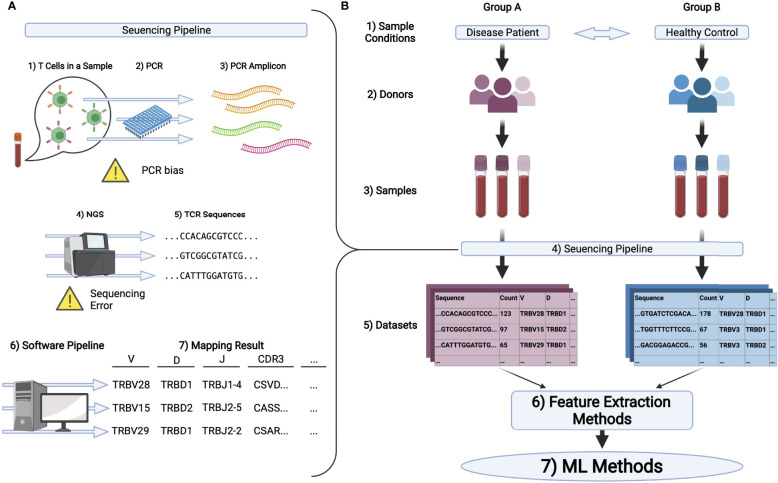
**(A)** Schematic illustration of the pipeline in TCR repertoire analysis. 1) First, T cells in samples, typically being collected from peripheral blood, are processed to extract its DNA or RNA of TCRs. 2,3) PCR is conducted to amplify the signal. 4,5) Then, the amplified DNA or cDNA is sequenced by NGS to obtain TCR sequences. 6,7) Finally, these sequences are mapped to the reference genes by the software pipeline introduced in the main text and analyzed further. **(B)** A typical experimental flow for applying ML methods on repertoire datasets. 1-3) Samples are collected from multiple groups of donors who have different immunological and physiological conditions. 4,5) By the pipeline illustrated in **(A)**, the dataset is obtained for each sample typically in the format of a table or matrix. 6) Datasets are encoded to ML friendly formats (feature vectors) using feature extraction methods. In bioinformatics, it is common to analyze gene expression matrices, which summarize the expression level of each gene for each sample. In repertoire analysis, for each sample, we have a matrix, each raw of which represents the sequence of one TCR, its observation count, its gene usage, and other properties of the TCR. Note that typically 10^4^ to 10^5^ different sequences are observed per sample and that only a limited number of overlapping sequences are usually detected among samples. Therefore, a relatively large sparse matrix must be handled for repertoire analysis. 7) ML algorithms are performed on the encoded datasets.

We can quantify TCR repertoires through repertoire sequencing (AIRR-seq) using NGSs. A typical repertoire sequencing procedure is summarized in **Figure 1A**. Samples such as peripheral blood mononuclear cells (PBMC) are collected, and their CDR regions are amplified by polymerase chain reaction (PCR). Then NGSs are used to read the amplified sequences. As CDR3 is the most diversified region in the TCR gene, many protocols are developed for CDR3 sequencing (29, 30).

Repertoire sequencing is one of the most actively developed sequencing technologies. In addition to the conventional procedure described above, single-cell repertoire sequencing has also been developed in recent years (4, 31). Using such protocols, for example, the pairing of the TCR*α* and TCR*β* chains can be measured (4, 31). Furthermore, dual TCRs can be investigated (32–36). As this review is primarily dedicated to repertoire analysis methods, we focus mainly on the potential biases in the conventional sequencing methods, which may skew the results of the ML methods.

First, PCR introduces various biases originating from the amplification. The sequence composition influences the amplification ratio of PCR. Multiple primers are also a source of biases. Multiple primers are commonly used in repertoire sequencing (37) because the edges of the CDR3 region are diverse depending on the choice of V (and J) genes. These primers are designed for known V (and J) genes. As a result, CDR3 sequences composed of unknown V (and J) alleles may not be amplified (30). In addition, multiplex PCR is also influenced by the amplification bias (30). Such quantitative bias affects a variety of ML methods introduced later. For example, diversity-based methods in observation frequency-based methods can be directly skewed. Various proliferated clonotype discovery methods are also affected.

Second, PCR and NGS introduce errors in the TCR sequences (29, 38, 39). It is estimated that about 2% of the PCR amplicons contain some sequencing errors in TCR sequencing (40), and 1-6% of sequences yielded by NGSs (Illumina) are erroneous. Erroneous sequences lead to false-positive clusters and skew the diversity in observation frequency-based methods. In contrast, dissimilarity-based methods aggregate similar sequences into a cluster. Therefore, they can be less affected by such errors.

Third, the starting material matters. We can employ either DNA or RNA of TCRs. In general, DNA-based methods are supposed to be more quantitative than RNA-based ones, as the number of RNA copies fluctuates among cells (30). However, a recent systematic review (41) suggests that the starting material may not always be the determinant of correctness or sensitivity. Moreover, RNA-based methods have some qualitative advantages. For example, some RNA-based protocols can capture the full-length TCR sequence, which contains CDR1,2 and 3 (30).

Many protocols have already been proposed to reduce such biases and errors. Certainly, their magnitudes can differ by protocol (29, 38). However, each method have both advantages and disadvantages. To apply ML methods to any data, we need to mind the protocol used to derive the data and be aware of the introduced bias beforehand. The following reviews are referred for details of each protocol (30, 31, 37).

Moreover, repertoire sequencing is affected by various batch effects. As we reviewed in this section, the choice of experimental protocol affects the result. Even if the protocol is the same, various conditions, such as different batches and different facilities where samples are collected, can be distinguished by ML algorithms (42, 43). These batch effects can be problematic in applying machine learning because of shortcut learning (44). We here adopt a famous example from medical image processing to intuitively explain the concept of shortcut learning. In the pneumonia detection task from an X-ray image, the performance of ML models is known to be dropped if tested by the datasets from other hospitals. It was revealed that ML models seemed to distinguish from which hospital an image was taken (45). As every hospital has different pneumonia prevalence rates, the model outputs positive if the sample seemed to be taken in a hospital with a high prevalence rate and can achieve a decent performance score. However, of course, if an image is not taken at the known hospitals, the model cannot answer correctly. In this situation, the hospital classification task was easier and was thereby used as a “shortcut” for the pneumonia detection task. As ML can distinguish various experimental conditions because of the batch effects, shortcut learning can also happen in ML-based repertoire analysis. There are attempts to remove the batch effects in repertoire sequencing. Some of errors and biases can be corrected by bioinformatic post-processing (29, 38, 40, 46). Such algorithms are implemented in popular software such as MiXCR (47). They are successful in reducing errors and biases (40). However, we have to be aware that the batch effects may not always be corrected. Thus, we must be careful when applying machine learning methods to repertoire datasets. For detailed comparisons of software, refer to (40, 46).

### Current Pipeline and Datasets for TCR Repertoire Analysis

Currently, a variety of TCR repertoire datasets are available to the public. There are two main types of platform hosting repertoire datasets. The first one is a public database, Sequence Read Archive (SRA)^2^, to which we can register raw sequences (e.g., FASTQ files). To download data, users need to find the accession number of International Nucleotide Sequence Databases (INSD)^3^ and use software such as sra-toolkit^4^. Each read sequence in a FASTQ file generated by NGSs is mapped to the reference sequences to annotate CDRs and selected V(D)J genes. Several pipeline tools for the analysis of FASTQ files have been proposed and developed, among which IMGT/HighV-QUEST (48), igBLAST (49), and MiXCR (47) are popularly used in previous studies. For performance comparisons of the major tools, we refer the reader to these review articles (50, 51). This workflow is summarized in **Figure 1A**.

The other is the platforms dedicated to immunosequencing datasets. For example, VDJServer^5^ (52) and immuneAccess^6^ have been widely used in recent years. Once FASTQ files are uploaded to these services, they will automatically process the files, and various analyses can be performed on the web. Such services seem to be highly appreciated by emancipating users from setting up a local environment or being bothered by complex software options. Still, there is no de facto standard for such repositories, and this has led to the development of curated databases such as iReceptor^7^ (53) and TCRdb^8^ (54) for scattered datasets.

To efficiently collect information on TCR repertoire analysis, it is also recommended to use other major repositories and communities as follows; VDJdb (55), a database that combines information on TCRs, antigens, and MHCs; Immune Epitope Database (IEDB) (56), a database of immune epitopes; McPAS-TCR (57), a database that organizes and collects TCR sequences related to various pathogens; and Adaptive Immune Receptor Repertoire (AIRR) community (58), a community for sharing antigen and repertoire datasets.

### Challenges in TCR Repertoire Analysis

In general, the basic approach for extracting useful information by comparing samples with others of different conditions is to contrast the information shared or not shared between samples in the same condition or those across different conditions like **Figure 1B**. In TCR repertoire analysis, the TCR sequences commonly observed among different individuals (called public TCR sequences) are considered important (59). However, due to the diversity of TCR repertoires, the number of public TCR sequences is very small compared to the total number of sequences observed in each sample. Therefore, they may not be sufficient to characterize the differences among the sample groups.

In addition, for sequences observed uniquely in each sample, it is not easy to distinguish whether they are attributed to the differences of individuals or to those of sample conditions such as abnormalities or diseases. The difficulty of associating observed sequences with sample conditions is one of the major problems in repertoire analysis due to the diversity of TCRs. Moreover, the TCR repertoire may change over time due to the donor’s health history such as injury, infection, and aging (1). Thus, the individual difference in TCR repertoire is large. Therefore, we should perform the analysis by taking into account not only the current but also the past health condition of the donors.

Furthermore, T cells isolated from peripheral blood samples are commonly used to measure human TCR repertoire. From each sample, we typically measure TCRs of about 10^4^ to 10^6^ T cells, which is a tiny fraction of the donor’s approximately 10^11^ Tcells (60, 61). Thus, quantitative analysis of TCR repertoire has its own difficulties due to the diversity and chronological variation of TCR repertoire and also to the high population size of T cells compared with the measurable size.

Moreover, we still cannot directly know what antigens a specific TCR sequence recognizes. Therefore, only from sequence information, we cannot compare or measure the similarity of TCRs by their antigen recognition profile. Experimental analyses of antigen-specific TCRs are widely performed (62–64), but they cannot be exhaustive, and we cannot conduct such analysis on every TCR. Although we have TCR-pMHC binding prediction methods, some of which we review later, the performance is still limited. In addition, the diversity of TCRs, MHCs, and antigens makes it impractical to calculate the complete recognition profile. This is problematic because we may need to utilize similar but different sequences to compare or characterize repertoires, as the number of shared identical TCRs is very small because of the high variety of TCRs and the limited sample size we mentioned earlier. Although a lot of experimental evidence such as (64, 65) implies that similar TCR sequences may recognize similar antigens, there is no *a priori* similarity measure. We need to devise a new way to calculate the similarity of TCRs.

These challenges are not the only obstacles in TCR repertoire analysis for understanding the dynamics of TCR repertoire. As we saw earlier, experimental procedures for repertoire sequencing using PCR or NGS inevitably introduce batch effects and errors. Some of the software tools introduced in the previous section correct and debias the sequencing data to some extent. However, not all the errors and batch effects can be removed.

**Figure 2:**
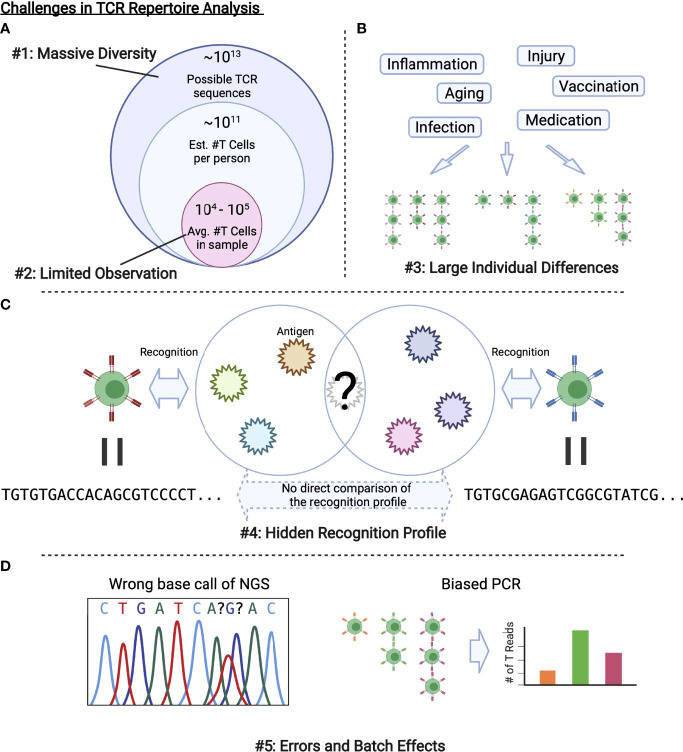
Challenges in TCR repertoire analysis. **(A)** Only limited observations are possible compared with the massive diversity of TCRs in a body or that of possible TCR sequences. **(B)** Various factors alter the repertoire, which results in large individual differences. **(C)** As we cannot observe all the antigens that a TCR recognizes, we cannot directly evaluate the similarity between TCRs with different sequences. **(D)** Experimental procedures including PCR and NGS inevitably introduce errors and batch effects.

**Figure 3:**
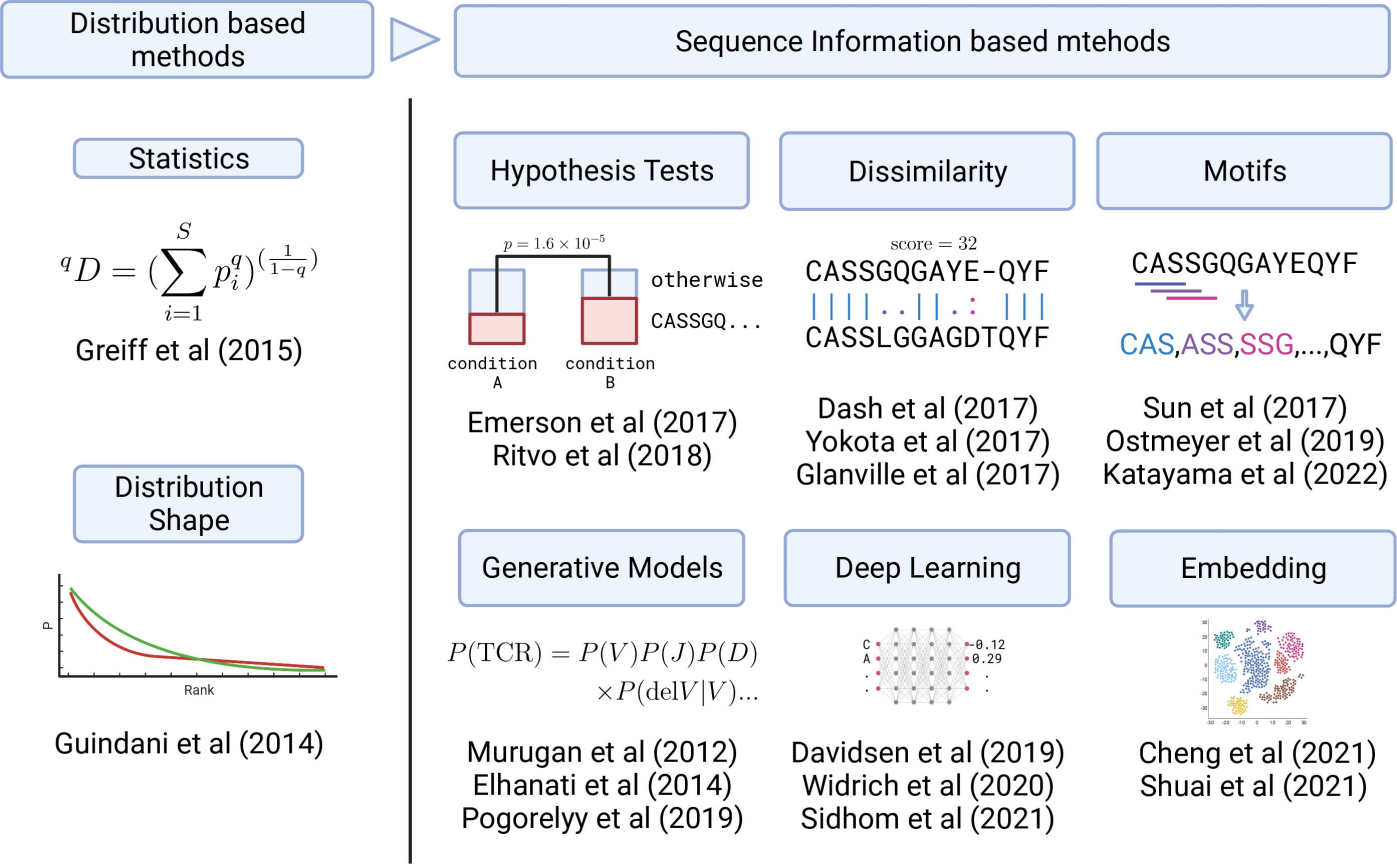
Graphical summary of the development in TCR analysis methods. Early analysis was based on the TCR clonotype abundance (frequency) distribution (left panel). Recently, sequence information has started to be utilized in various ways (right panel) by employing statistical, ML, and DL methods.

These problems summarized in **Figure 2** are related to the development of bioinformatic and ML methods to be introduced in the next section. Each method approaches to these challenges in a unique way, which can be categorized as in **Figure 3**. In the following sections, we review each category one by one.

## Observation Frequency Based Methods

In the conventional analysis of TCR repertoires, statistical indices from ecology have been employed. In ecology, the complexity of ecosystems has been measured by diversity (66). Typically, diversity is calculated by the rarity weighted count of the species. If a species has a dominant population, the diversity of the ecosystem is small. In contrast, if there are many rare species, the diversity increases.

By treating each TCR clonotype as a species, diversity can be measured for a TCR repertoire in a sample. In immunology, the diversity of TCR repertoire is closely related to the clonal expansion (an increase in the proportion of T cells with the same TCR clonotype caused by a proliferation of T cells, which decreases the diversity of TCR repertoire) against specific antigens (1). By applying the species diversity analysis methods in ecology, the degree of clonal expansion has been associated with various sample conditions. A typical example is an approach to quantify the diversity of amino acid sequences in CDR3 using indices such as Hill’s number (67). Around 2010, the quantification of the diversity of TCR repertoires using probability models were proposed (68), which enabled us to characterize differences between samples. Both Guindani et al. (69) and Rempala et al. (70) employed the Poisson abundance model (68), to not only fitting the abundance distribution shapes, but also to classify the samples using the estimated parameters of each sample. This approach is still being investigated: PowerTCR (71) proposed a probabilistic model not based on Poisson abundance model in 2018.

However, these approaches employ only frequency information and do not directly utilize the sequence information. As a result, important information can be obscured or lost. For example, even if the samples have very similar frequency distributions, the sequences observed at high frequencies might be completely different. In particular, it is difficult to examine or identify particular sequences that caused the differences between samples only from the frequency information, which is important for practical applications. Moreover, utilizing ML methods enabled the processing of sequence information without compressing it down to the frequency information. Therefore, the recent advances in TCR repertoire analysis have occurred primarily in sequence-information-based methods using ML methods. We categorized them by their approaches, as summarized in **Figure 3**, and will review each of them in the following sections. Nevertheless, frequency-based methods are still practically effective for small datasets as the frequency distribution can be obtained relatively stably even from a sample containing a small number of TCRs.

## Utilization of Sequence Information

As we saw in the previous section, frequency distribution based methods can only provide the degree of difference between different samples. In particular, specific sequences characterizing the sample differences are of particular importance. Since we are interested in specific sequences characterizing the sample differences, we need another approach that can directly utilize sequence information to identify those specific sequences. One of the most illustrative and important applications of sequence information is monitoring minimum residual disease (MRD), a kind of T(B)-cell leukemia (72). As dominant T(B) cell clones themselves are the direct cause of MRD, unusually proliferated TCRs (BCRs) can be utilized as biomarkers to monitor the progression of the disease. In monoclonal leukemia, the identification of such dominant sequences is fairly easy because the dominant clone sometimes occupies more than 75% of the T cells (73).

However, we may not be able to find such obvious sequences for other diseases or conditions. Unlike leukemia, the most abundant clone in a sample may not be related to diseases or conditions. We must find a portion of the sequences shared between the samples of the same condition, but this is not straightforward. Due to the diversity and individual differences of TCR repertoires, the number of shared sequences is typically very small. Even if we find shared TCRs, we must statistically discriminate whether such shared TCRs are yielded by a condition or by chance. It should be noted that we need to devise a way to evaluate “similar” sequences because we cannot directly observe the similarity of TCRs. To overcome these problems, ML has been utilized. In this section, we review three major categories of methods, which are based on hypothesis tests, dissimilarity, and motifs, respectively.

### Hypothesis Test Based Methods

One of the straightforward ways to extract relationships of specific TCRs and sample conditions is to use hypothesis tests to judge whether the number of observed TCRs is significantly large or small for a specific sample condition. For example, Emerson et al. (16) collected peripheral blood samples from a total of 641 donors, 289 affected and 352 unaffected by CMV, and identified the TCR*β* sequences specific to the CMV-affected donors using Fisher’s exact test. In addition, by utilizing the identified 164 CMV-specific TCR*β* sequences as features of a repertoire, they designed a discriminative model of beta-binomial distribution for predicting CMV infection. De Neuter et al. (74) replicated the results on another dataset and showed that a random forest classifier using the observation counts of these TCRs in a sample also works well to predict the infection. Emerson’s method, however, ignores sequence similarity completely and only utilizes the information of “Public TCRs.”

In contrast, Ritvo et al. (75) proposed a method called TCRNET, which utilizes sequence similarity to estimate clusters of similar TCRs that are significantly proliferated in specific samples. Here, similar TCRs are defined as those derived from the same V and J genes and differ at most by one amino acid sequence. Then, the number of TCRs in the target cluster is contrasted with the number of TCRs with the same V, J genes and CDR3 sequence length as the target cluster. If the proportion is found to be significantly larger in a specific sample by the binomial test, the target cluster is judged as a proliferated cluster.

These methods require counting the same or similar TCRs. This process is very slow because, in a naive implementation, every possible pair of sequences must be compared. CompAIRR^9^ is developed for a faster exact or approximate search for shared TCRs.

### Dissimilarity Based Methods

The methods introduced above compare only a specific TCR or a cluster of TCRs with the others. Therefore, they abandon the sequence information of the others, even though they constitute most of the sequences in samples. Some methods have been proposed to exploit such information. In particular, we review the methods based on the dissimilarity between TCRs. Network analysis based on sequence similarity has been used for a long time. For example, classification of healthy and leukemic samples is performed on the BCR sequence network of each sample in which all sequences differ at most one residue are connected (76). In TCR, a similar network analysis revealed the public TCRs conserved between mice and humans (77).

More complex dissimilarity indices tailored for TCR analysis have been proposed. Dash et al. (78) quantified the differences between two TCR sequences by weighted Hamming distances and visualized epitope-specific TCR clusters by dimensionality reduction and clustering of their dissimilarity matrices. Their method, called TCRdist (78), has become a popular method to search for epitope-specific TCR sequences. TCRdist focuses mostly on evaluating the differences of TCRs. By contrast, RECOLD (79), which was proposed by our group, is designed to measure the differences between samples. RECOLD calculates the distance between all the observed sequences in all samples to create a dissimilarity matrix. Then, dimensionality reduction is performed on the matrix, and every observed sequence is embedded in a shared low-dimensional space as a point. In this space, each sample is represented as a probability distribution, and the difference between samples is quantified as the difference of distributions by Jensen-Shannon Divergence. In addition, RECOLD can identify the sequences specifically contributing to the differences of samples using the bootstrap method.

New methods based on TCR-level dissimilarity are still actively and continuously explored. A method called GLIPH (63) integrates sequence information and observed frequency information with CDR3 length and HLA to estimate epitope-specific sequences. iSMART (80) and GLIPH2 (81) have been released recently to improve the performance and the applicable data size. In TCRdist3 (82), TCRdist-based distance can be combined with motifs (introduced in the next section) to characterize TCR clusters.

On the other hand, some methods are devised for calculating the distances between repertoires directly. Repertoire Dissimilarity Index (RDI) (83) compares the usage of V(D)J gene segment. ImmuneREF^10^ utilizes various interpretable indices such as diversity indices and positional amino acid frequencies.

As in the case of the hypothesis-based methods, the computation cost is important for dissimilarity-based methods, which also perform a lot of sequence comparison. ClusTCR (84) achieves faster clustering by focusing CDR3 and compromising flexibility in the sequence alignment. GIANA (85) used a different approach. In GIANA, a lightweight linear transformation equivalent to sequence alignment on BLOSUM62 is constructed. Then, every sequence can be encoded into a coordinate in the euclidian space, where clustering is fast.

### Motif Based Methods

The dissimilarity based methods characterize TCRs (or samples) by the relative distances between them. Alternatively, we can directly encode TCRs (or samples) into feature vectors and apply ML methods to the vectors. A conventional but effective method to create such feature vectors is the k-mer method. It characterizes a TCR (or a sample) by the observed frequency of all possible k consecutive substrings (motifs) in the sequence (or sequences in the sample). Therefore, in a typical 3-mer method, its feature vector has approximately 21^3^ dimensions (21 = 20 human amino acids + a symbol representing the edges of the amino-acid sequences). The k-mer features have been combined with various ML methods: LP-boost (86); Bayesian discriminators (87); and SVMs (87). They were applied to TCR *β*-chain CDR3 datasets to discriminate whether a sample had been treated with ovalbumin or not, which was used to stimulate immune responses. We also proposed MotifBoost (43), which merges the k-mer encoding and Gradient Boosting Decision Tree (GBDT) (88) for repertoire classification. Along with proposing a new method, we also investigated the nature of the k-mer encoding and revealed that CMV infected and healthy samples are well separated in the k-mer feature space derived by a PCA-like unsupervised learning method called Gaussian Process Latent Variable Model (GPLVM) (89). This result indicates that the k-mer encoding can naturally capture the intrinsic characteristics of repertoires. Moreover, k-mer based methods work effectively even on smaller samples compared to the other methods.

As we mentioned earlier, we still do not fully understand what kind of factors determine the similarity between different TCRs. However, the success of the dissimilarity-based methods, which is based on the hypothesis that similar TCRs work similarly in the body, implies that the hypothesis is true to some extent. Moreover, the success of k-mer encoding support and strengthen the view that some important motifs play a central role in determining the similarity of TCRs. This is also supported by the fact that shared motifs of antigen-specific TCRs are found in various conditions (62–64).

While being conventional, k-mer encoding and combined ML methods still have room for further improvement and development. For example, Ostmeyer et al. (90) combined the 4-mer method with logistic regression to discriminate between cancerous and healthy tissues. In this work, feature vectors are created differently from the conventional way. Each 4-mer motif is represented as a 20-dimensional vector consisting of four 5-dimensional biophysicochemical feature vectors of each amino acid. Therefore, a TCR is converted into a bag of 4-mer feature vectors. To deal with this setup, they employed the multiple instance learning framework. Specifically, they trained a logistic regression model to assign a score, which is the probability that the motif is related to cancer, for each motif. A sample’s score, which is used for sample-level classification, is defined as the maximum score of the motifs found in the sample.

As a good representation of data is decisive in ML, we expect that more applications appear, which are built around k-mer methods or other data representation methods.

## Application of Generative Models

Most of the methods mentioned above are used for characterizing the differences between samples. Thus, they usually compare samples obtained from different conditions by assuming that the dataset to be analyzed is from a cross-sectional or longitudinal study. However, careful effort is required for obtaining datasets from multiple experiments. Recruiting a sufficient number of donors for every sample condition is difficult, especially if they are rare.

To solve this problem, methods based on generative models have recently been explored. These methods employ mathematical models for the generation of TCRs, which have been intensively developed since 2012. For TCR generation in the thymus, a probabilistic model implementing the biological mechanism of V(D)J recombination was proposed (91). Various extensions to this model, especially for inference methods, have been proposed based on Monte Carlo simulation (92), improved expectation maximization (EM) algorithm (93), and dynamic programming (94). For TCR selection in the periphery, Elhanati et al. (95) devised another probabilistic model. Their model employs the actual peripheral repertoire dataset to estimate the probability distribution of post-selected TCRs, and utilizes the TCR generation model of (91) to infer that of unobserved pre-selected TCRs. This model is trained to predict the difference between the two distributions.

Based on the same idea of substituting the unobserved datasets with a generative model, Pogorelyy et al. (96) developed a method called Antigen-specific Lymphocyte Identification by Clustering of Expanded sequences (ALICE), which can characterize samples obtained from only one condition by contrasting them with the sequences generated by a generative model as reference repertoires. This strategy is also applied to characterizing TCRs (92).

The generative model can pave the way to quantify the abnormality of a sample and to infer its responsible sequences only from a snapshot sampling of the patient’s repertoire, without expensive effort to conduct cohort studies. However, challenges remain for its practical and reliable employment. For example, because the TCR generation model utilized in ALICE does not take into account the individual difference that affects the TCR repertoire [e.g., genetic background (97) and age (26)], the parameters of the generative model may need to be adjusted to the conditions of individual samples to further enhance its reliability.

### Simulation of Repertoire

The advance of generative models leads to the emergence of some simulation software, which create pseudo repertoire datasets. Simulated datasets have been used to assess the performance of repertoire analysis methods. For example, a simulated dataset was used to assess the performance of the V(D)J genes identification for B cells (98).

IgSimulator (99) is one of the earliest repertoire dataset simulators. AbSim (100) simulates the temporal development of mutations in B cells. However, these simulators were made for antibody sequences, not TCR sequences. ImmuneSIM (101) is capable of simulating TCR repertoires. In addition, its remarkable feature is the simulation of repertoires for classification. It can implant k-mer like sequences into the repertoire dataset. Classification methods can be tested whether they can find the implanted TCR or repertoire or the implanted motif itself. As motifs play an important role in characterizing repertoires (see motif-based methods), k-mer like signal implanting is recently adopted in some studies (102, 103).

Using simulation, further evaluation of analysis methods can be performed. For example, the classification performance was evaluated in various conditions with different density of signal, sample sizes and so on as done in (103). Evaluations like this cannot be conducted using only real datasets.

## Application of Deep Learning

Deep learning (DL) is a class of ML algorithm, which achieves good performance in various fields. DL has been pervading various areas of biology such as genomics (104) and systems biology (105), and it has also recently been applied to repertoire analysis. Again, DL itself is just another ML algorithm. However, representation learning, which is one of the notable features of deep learning, allow DL models to achieve high performance by learning appropriate representations from data without explicitly providing the mechanism behind it (106). On the other hand, most of the models we introduced earlier used hand-crafted features or were based on the human knowledge. We call such models “hand-crafted model” hereafter. While the generative model of TCRs introduced above is a hand-crafted model that explicitly implements biological mechanisms such as V(D)J recombination, Davidsen et al. (107) proposed a Variational Auto Encoder (VAE) (108) based generative model that treats the TCR generation like a string generation task. Another feature of DL is that representations learned in one task can be easily transferred to other tasks [called transfer learning (109)]. DeepTCR (110) solves classification problems using features obtained from a VAE-based generative model.

Not only generative models like VAE but also discriminative models are utilized for repertoire analysis. For example, DeepRC (102) utilized a popular class of DL model architecture called attention mechanism for the repertoire classification problem. Simply put, the attention mechanism is a kind of learnable weighted average (111). DeepRC encodes each amino acid sequence in the repertoire to a vector and analyzes its importance through the attention mechanism. Classification is made on the weighted average of the encoded vectors.

DL is also being intensively applied to the prediction of affinity between pairs of T cells and antigens (112, 113), as well as triplets including MHCs (114). TCR-pMHC binding prediction task is one of the most actively studied topics in immunoinformatics (115). The task is to predict whether or not the target antigen will be recognized by a TCR using the sequence information of the TCR and the antigen protein. As Alphafold2 (116) has made an innovation in predicting the structure of proteins from their amino acid sequences, DL is expected to make a breakthrough in this area.

At this stage, DL-based methods have not yet demonstrated the performance to dominate hand-crafted models, in which human crafts the feature or the model structure, in this field. For example, a comparison between a hand-crafted generative model (95) and Davidsen’s VAE-based generative model (107) was conducted (117). This paper concluded that the hand-crafted model outperforms DL-based models with lower computational cost and higher interpretability. For peptide-MHC binding prediction, according to a systematic performance comparison review conducted in 2020, ML-based models still scored better than DL-based models on average (118). In addition, our group compared a DL model and ML models by changing the available data size for learning on a repertoire classification task and found that the performance of the DL-based model deteriorates on the small datasets (43).

According to the current trend, the application of DL in this field will be investigated even more intensively in the future. For example, some more recent DL-based peptide-MHC methods reviewed in the next section are showing better performance than the traditional methods on some specific datasets. However, DL may not wipe out the need for traditional biological hand-crafted models because of its expensive computation cost, lack of interpretability, and data-intensive nature. Instead, the integration of hand-crafted and DL-based models is being explored. In a recently proposed model for T cell selection called soNNia (119), a hand-crafted generative model for TCR generation probability (95), which was used for comparison in (117), is combined with a DL model of the TCR selection. For TCR-pMHC interaction prediction, a combination of DL and traditional ML methods is also being pursued (120).

### Embedding Methods Based on Representation Learning

In the recent advances in Natural Language Processing (NLP), self-supervised representation learning draws attention, which utilizes the nature of data as a target signal to learn good representations. This is realized by the ability of DL to acquire good representations mentioned in the previous section. One of the earliest successful approaches is Word2Vec (121), which encodes a word to a numeric vector (Word Embedding). In a Word2Vec training method called CBOW (continuous bag of words), a neural network (NN) that converts a word to a vector is trained to predict a masked word in a sentence using encoded vectors of its surrounding words (122). Word2Vec is utilized widely to convert textual data to numerical representation in NLP and also is applied to repertoire analysis. Immune2Vec (123) is inspired by Word2Vec and treats a TCR/BCR as a sentence and a k-mer as a word, respectively. Representation of a TCR/BCR, which is composed of many k-mers, is derived by averaging all k-mer vectors, which is a similar procedure to FastText (124) in NLP.

After the success of Word2Vec, various NN architectures for self-supervised representation learning in NLP are developed. One of the noticeable approaches is neural language models. A language model is a generative model to predict words from the context. CBOW is a representative example which predicts a word from context words. Thanks to the invention of a new NN building block called Transformer (125), which utilized the attention mechanism we mentioned in the previous section. NNs can handle more distant dependencies in a text. New neural language models like BERT (126) exploited the Transformer’s ability and broke the former models’ records in various tasks. These models are trained to predict a masked word similarly to CBOW. However, in contrast to CBOW, they can predict one or more meaningful sentences, not a word. One such language model called GPT-3 can write natural texts, e.g., news articles (127). We can also utilize a neural language model to embed a sentence using the output of the hidden layer (Sentence Embedding). Such sentence embedding is revealed to be a very good representation and can be applied to multiple downstream tasks in NLP, from question answering to translation, with little additional training for each task (called fine-tuning) (128). Training of the language model itself (called pretraining) requires a large corpus and enormous computation resources. However, once the training is done, the same model can be applied to various problems with fine-tuning using little data.

Language models have also been employed in repertoire analysis. Before that, language models have been intensively applied to general protein sequences (129–132). BERTMHC (133) showed utilizing the pre-trained model of (129) actually increases the performance in the peptide-MHC (Class II) binding prediction task. ImmunoBERT (134) used the same pre-trained model for the peptide-MHC (Class I) binding prediction task. Hashemi et al. (135) employed the pre-trained model of (131) and fine-tuned them for peptide-MHC (Class I) binding prediction and achieved higher performance compared to a previous software. Some papers perform pre-training on their own on the repertoire sequencing dataset. In Leem et al. (136), each amino acid in a TCR is treated as a word, and a TCR is treated as a sentence to pre-train a BERT language model (AntiBERTa). AntiBERTa achieved a higher ROC-AUC in a paratope prediction task than other tools.

The utilization of language models is not limited to embedding. In Shuai et al. (137), another language model called GPT-2 (128) is utilized for pretraing on an antibody generation model (IgLM). Because GPT-2 is designed for full sentence generation, unlike BERT, IgLM can generate new antibodies (CDRs). A new antibody design workflow is proposed in the paper and outlined as follows: First, many antibodies are created using IgLM. Then the3D structure for each antibody is calculated. Finally, the properties of the generated structures are computed to select better antibody candidates.

## Machine Learning for Repertoire Analysis in Practice

In this review, we focused mainly on the technical aspects of ML and DL methods and categorized them by their approach. As a result, we cannot cover all topics, especially those being relevant to practical applications. This may be compensated by a thorough review of the repertoire analysis methods before 2019 in (138), and another review that introduce many methods categorized by task (139). In addition, more ML applications can be found on the pMHC-epitope analysis in (140–143), and on longitudinal analysis in (144, 145).

To practice ML methods, we can refer to the author’s implementation in most cases. We can find a comprehensive list of such implementations and other software in (146). In addition, there exist some libraries that implement multiple popular methods to be used for general analysis. In particular, VDJTools (42) and tcR (147) (Immunearch^11^ is its successor) are equipped with a broad range of basic analysis methods and are widely used in practice. Moreover, new libraries are being developed such as ImmuneML (148), which focuses more on ML methods.

As for the topics that those sources cannot fully cover, we discuss the following two topics in relation to the practice of ML methods in TCR repertoire analysis: One is prospective practical applications of repertoire analysis, such as blood testing and cancer vaccination. The other is repertoire analysis of COVID-19

### Applications of Repertoire Analysis

Recently, applications of repertoire analysis have been developed rapidly. One of the most prominent applications is blood testing (149). In this field, the diagnosis of MRD (see UTILIZATION OF SEQUENCE INFORMATION) and the COVID-19 testing (see the next section) are already approved by FDA. There are potentially more diseases that can be diagnosed by repertoire sequencing. For example, autoimmune diseases such as lupus erythematosus (150), rheumatoid arthritis (150), and lupus nephritis (151) have been successfully classified with the V-J gene usage distribution feature and a random forest classifier. In the BCR repertoire, IGHV gene selection was analyzed for multiple autoimmune diseases (152).

In relation to autoimmunity, repertoire analysis revealed the features common to self-reactive T cells. Hydrophobic residues (153, 154) or Cysteine (154) on CDR3 are related to their self-reactivity. Hydrophobic CDRs enrichment in regulatory T cells is replicated by a logistic regression model with 606 T cell features to predict whether a cell becomes a regulatory T cell or not (155). Prediction of self-reactive T cells may play an important role in the diagnosis of autoimmune diseases in the future.

Another prominent application is neoantigen vaccines to treat cancer. Neoantigen is a tumor-specific antigen that can be used to target tumor cells. Thus, neoantigen vaccines stimulate T cells to attack tumor cells. Neoantigen vaccines should be personalized because tumors of different individuals tend to acquire different mutations and express different neoantigens (156, 157). Repertoire analysis is expected to reduce the labor required for finding individual neoantigen (158). The finding of neoantigens *in silico* is typically performed as follows: First, tumor-specific mutations and their transcripted proteins are identified by sequencing. Second, from those proteins, all antigenic peptides that mark cancer cells are listed. Third, the peptides that can bind to the patient’s MHC well are screened. Finally, the obtained peptides are tested to determine whether the pMHC complex can be recognized by T cells or not. Repertoire analysis is used in the third step to predict the affinity of peptide and personal MHC. A couple of software was published for this task (118). On the other hand, immunopeptidome is studied as a different approach to find neoantigens (159). This approach is also interesting in relation to repertoire analysis. In this approach, TCR-pMHC complexes in tumor tissues are collected and analyzed to retrieve their peptide sequences. As the peptides are already assured to bind to MHC, we can skip some of the described screening process. Immunopeptidome can be seen as a peptide repertoire, and its analysis might provide insight into TCR repertoire in the future.

We reviewed some potential applications of repertoire analysis in this section. To realize such applications, we need reproducible and robust results. For clinical applications, standardized protocols must be established. For example, a standard experimental protocol is proposed for MRD diagnosis (160). Also, bioinformatic pipelines are not yet standardized. We will expect more standardized workflows to appear in the future. An example is a new standard format for repertoire dataset proposed by AIRR Community (161).

### Repertoire Analysis for COVID-19

Understanding COVID-19 has been one of the most important research topics in recent years, and repertoire analysis has revealed various characteristics of COVID-19 so far. In this section, we will see how the ML-based repertoire analysis introduced in this review is used in the COVID-19 study.

Repertoire analysis has been employed to investigate the nature of COVID-19 infection. Most basic observation is the change in diveristy. Many studies reported the low TCR repertoire diversity in active COVID-19 patients (162–165). Some studies further reported that the severity of the symptom is related to the lower diversity (163, 166). However, it should be noted that decrease in TCR diversity is not necessarily specific to COVID-19 infection but common to various virus infections (164). Cheng et al. (167) investigated V(D)J gene usage and found that some V*β* genes, which are estimated to have a high affinity to SARS-Cov2 spike protein antigen, were enriched in severe COVID-19 patients.

Further insights are also provided by using sequence information based ML methods. In Simnica et al. (168), COVID-19 public TCRs are investigated. GLIPH2 (81), one of the dissimilarity-based methods we reviewed, was used to cluster TCRs and select COVID-19 related TCRs by Student’s T-test (similar to Emerson et al. (16) introduced as one of the hypothesis test based methods). GLIPH2 was also employed in Chang et al. (166) to characterize the TCRs related to the severity of the symptoms. Minervina et al. (169) also examined the dynamics of COVID-19 patients’ repertoires over time using the hypothesis test previously proposed by the same group (170) to distinguish proliferating clones. Quiros-Fernandez et al. (171) revealed the cross-reactivity of CD8+ T cells in unexposed donors to the COVID-19 epitope, which is derived using NetCTLPan (172), an NN-based peptide-MHC binding prediction software.

We cannot cover all the COVID-19 related literature here. For further reading, see (173) for early researches and (174) for recent updates. For repertoire diversity and COVID-19, see (175, 176). Note that, as COVID-19 is still not fully understood, these results should be further validated in the future.

As a practical application, the repertoire analysis is utilized to diagnose COVID-19. Adaptive Biotechnologies, a US-listed company, applied the ML algorithm that they developed for CMV [in Emerson et al. (16), introduced in Section 3.1] to the COVID-19 dataset. It was demonstrated that the algorithm successfully distinguished the sample’s COVID-19 infection status (6). Adaptive Biotechnologies received EUA (Emergency Use Authorization) for the COVID-19 test from the FDA. Nevertheless, repertoire-based test may not be the first choice for COVID-19 diagnosis. First, T(B)CR repertoire can not provide direct evidence of SARS Cov-2 virus existence. Second, repertoire-based test requires sequencing, which costs substantially more than PCR or antibody tests. However, repertoire analysis can potentially reveal far more information than such tests (149), and the sequencing cost is decreasing. Therefore, in the future, repertoire-based blood testing can be utilized further (149).

### Small Sample Problem of Repertoire Datasets

The size of datasets is the major determinant of the performance of methods and the reliability of their results (43, 103). Therefore, the establishment and development of sufficiently large datasets are important equally to or even more than the development of analysis methods.

TCRdb (54), one of the major databases of TCR repertoire, contains 131 projects with a total of 8,341 samples of public datasets aggregated from various repositories as of November 2021. Since one project is usually associated with one paper, a rough estimate indicates that one paper contains 64 samples on average. In general, this number is considered small for applying ML algorithms, and actually, the classification methods mentioned above do not always work satisfactorily in some different classification tasks, especially when the sample size is less than 100 (43). A simulation also indicates that the number of samples affects the classification performance (103).

This situation is gradually changing with the appearance of large datasets containing several hundred samples, such as the CMV dataset in Emerson et al. (16). In addition, Adaptive Biotechnologies and Microsoft released a new COVID-19 dataset with 1,486 samples, one of the largest released ever as a single dataset^12^, which was used in (6). However, such a large dataset is exceptional, especially as that of the human repertoire, in light of the difficulty to collect a large number of patients with the same condition, e.g., infection records. Even though the number of publicly available datasets have been grown steadily (177), and will continue to grow, the small data size problem may not be readily resolved. Note that we might employ other animals’ datasets for some basic research (77). In VDJdb (177), datasets of mice and macaques are recorded. However, the number of the dataset is much fewer than that of humans.

Simulations are not only a powerful tool for repertoire analysis, as we saw earlier, but also can contribute to overcoming the situation, as generative models can create an unlimited amount of pseudo datasets. However, the employment of simulations in repertoire analysis may not always be assured, depending on the tasks and situations. For example, simulated datasets for repertoire classification tasks are created by embedding specific k-mer like signals only in repertoires belonging to specific classes (101–103). Though we know such motifs are important to characterize the binding property of TCR (63), other signals may be still missing. Also, each disease may affect repertoire uniquely [e.g., the difference between CMV and varicella zoster virus (VZV) (178)]. Therefore, until we have a plenty of real datasets, we can not know how we can characterize the changes in repertoire caused by a given condition. Therefore, we will still need real datasets, especially to enable new practical applications.

To alleviate the problem, we have to select appropriate methods for a given size of datasets, understand more about the limit of information that can be derived from a given data, and develop new methods that can integrate multiple datasets or work effectively even with small sizes of datasets.

## Discussion

In this paper, we have surveyed ML applications to TCR repertoire analysis by following its development from simple statistical indices to DL, as being summarized in **Figure 3**. For reader’s convenience, we summarized a detailed comparison between the methods in **Table 1**.

**Table 1:** Qualitative comparison of the methods reviewed in this article. In practice, both feature encoding methods and ML algorithms for specific tasks such as classification or regression are combined. As the choice of ML algorithms is usually arbitrary, this table is organized by the viewpoint of feature extraction.

Methods		Core Idea	TCR-level encoding	Repertoire-level encoding	ML methods combined with	Strength	Weakness	Notable Examples	Relationship with other methods
Distribution based models	Statistics (Diversity)	TCR diversity is related to healthiness and abnormality of immunological states. Diversity indices such as a rarity weighted count of TCR clonotypes can be used as basic parameters of the immunological state.	NA	A diversity index (a scalar value)	NA	Applicable to data with small sample size and/or small number of sequences.	Too simple and ignoring sequence information.	Grieff et al (67) used multiple diversity indices to create a repertoire-level feature vector.	NA
	Distribution Shape	The distribution of the clonotype frequency is used to analyze the structure of clonotype diversity. By fitting the sample distribution by probabilistic models, characteristic parameters of the distribution are estimated.	NA	Model parameters of distributions	Probablistic Model	Applicable to data with small sample size and/or small number of sequences. Flexibility of modeling.	Arbitrariness of modeling and ignoring sequence information.	Guidani et al (69) used a bayesian model to infer the number of clonotypes in a sample from the distribution.	NA
Sequence Information based methods	Hypothesis Test	The TCRs shared among the samples in a condition compared to others might be correlated with the condition. Such TCRs can be identified by hypothesis tests.	Significance of presence or absence of specific TCRs in a condition	A bool vector of the existence of the specific condition-related TCRs found by the hypothesis tests	Various Classifiers	Each TCR can be characterized by the relatedness to the conditions.	Ignoring most of the sequences.	Emerson et al (16) used a hypothesis-based method to find CMV-related TCRs and classify CMV infection based on the existence of such TCRs.s Ritvo et al (75) proposed a method to find proliferated clusters using a hypothesis test.	To include similarity, hypothesis tests are combined with dissimilarity-based methods (ex. Glanville et al., 63).
	Dissimilarity	Similar TCRs may play a similar role in the body. Distance between TCRs can be used to detect and cluster the similar TCRs.	Relative distance from other sequences. Manifold learning is sometimes used to calculate absolute position of the TCR in the latent space	Density distribution on the latent space	Clustering Algorithms and Manifold Learning	Utilizing all sequences to characterize samples. Each TCR is characterized by the relative distance from the other TCRs.	Computational cost of pairwise alignment.	Dash et al (78) used a dissimilarity matrix and visualized the epitope-specific clusters by manifold learning. Yokota et al (79) quantified the distance of repertoires by creating the inter-sample dissimilarity matrix. Glanville et al (63) integrate various information into the dissimilarity calculation (ex. length of CDR3)	NA
	Motif	Local patterns such as (k-mer) motifs in a TCR may be related to its function. Encoding TCRs by a vector of local features may be a good representation of TCRs.	Bag of k-mer. Atchley vector is also used to encode the TCR to more dense vector.	Bag of k-mer or aggreation of TCR-level encoding	Various Classifiers / Regressors	Utilize all sequences to characterize samples. Applicable to data with small sample size and/or small number of sequences. Each TCR is directly characterized as a feature vector.	Low flexibility in modeling.	Sun et al (86) used a 3-mer feature vector of each CDR3 and SVM for a repertoire classification task. Ostmeyer et al (90) used a 4-mer vector further encoded by the Atchley vector, which represents the physicochemical nature of amino acids. Katayama et al (43) applied a 3-mer feature vector to repertoire classification tasks on small datasets.	Motifs are sometimes used for calculating dissimilarity (ex. Mayer-Blackwell et al., 82).
	Generative Models	The mechanisms of generation and selection of TCRs are the determinants of TCR repertoire. Their modelling provides additional information to the observed and not-observed repertoires.	NA	Model parameters of the generative models	Probablistic Model and Manifold Learning	Utilizing all sequences to characterize samples. Applicable to data with small sample size and/or small number of sequences. Generation of pseudo data (for simulatiion, data augmentation etc.)	Validity of assumptions in models.	Murugan et al (91) modelled the biological V(D)J recombination process and used unselected TCRs to fit the model. Elhanati et al (95) modelled the thymic selection of TCRs and combined Murugan's model to estimate the parameter of the selection process. Pogorelyy et al (96) proposed a method to quantify the abnormality of repertoire using the generation probability from a generative model.	NA
	Deep Learning (DL)	Good representations of repertoires may be obtained by Deep learning and may improve the performance of various repertoire analysis	Various encoding based on VAE or language models (See embedding methods)	Inferred parameters of DL-based models	Generative Models and Embedding Methods	High flexibility in modeling. High performance if sufficient amount of data is provided.	Model is not explainable and data expensive.	Davidsen et al (107) proposed a VAE-based model to embed TCR sequences into the latent space. Widrich et al (102) proposed a Transformer-like model for a repertoire classification problem. Sidhom et al (110) used another VAE-based model to solve various regression/classification tasks.	Embedding Methods are closely related with DL.
	Embedding Methods	Because TCR sequences are a collection of strings, encoding TCRs to fixed-length dense vectors using NLP may lead to efficient algorithms.	Sentence embedding	NA	Various Algorithms incl. DL	High flexibility in modeling. Applicable to data with small sample size and/or small number of sequences (after pre-training).	Model is not explainable.	Cheng et al (166) employed a pre-trained general protein language model for the peptide-MHC binding prediction task. Shuai et al (137) performed pre-training using the repertoire sequence dataset (BCR) and measured the performance on a single downstream task.	NA

NA stands for not applicable.

Finally, we discuss the remaining technological challenges and outline the future directions in the development of TCR repertoire analysis. In particular, we focus on two topics, the small sample problem and the multimodal data integration.

The small sample problem of repertoire datasets we reviewed in the previous section is one of the most important problems that should be resolved in repertoire analysis. As mentioned earlier, the cost of large datasets will likely remain high. Thus, we need to address the problem by devising new analysis methods that can work on smaller but practical datasets. We have at least three representative approaches to achieve this goal. First, as we reviewed in the previous section, simulations can be used to create datasets. We expect more simulation software releases in the future. In this section, we discuss the other two approaches further.

Another possible direction is to utilize multiple datasets to solve a task. Two DL-based techniques which we mentioned earlier will play an important role to this end. Transfer learning can be employed to implement such a method. In transfer learning, we prepare a DL model that has already learned a good feature representation after training on a large unsupervised corpus, and then utilize it for feature extraction in the target task (179). This technique improves the performance of the target task, especially when the dataset for the target task is small. Similarly, representation learning is important. Good representations of repertoires may be learned from large amounts of unlabeled repertoire datasets. If such good representations are learned, classification of individual diseases, for example, may become possible with high performance even if only small amounts of data are available for the target diseases. As we saw earlier, this direction was already investigated using VAE (110). However, the size of the model is far smaller than those used in NLP, and the universality of the representation has not yet been discussed. Moreover, there is no standard task in repertoire analysis in contrast to NLP. Therefore, the models are not evaluated in terms of which downstream tasks can be applied *via* transfer learning. Recently, attempts appear, which utilize large language models in repertoire analysis (133–137, 180). In AntiBERTa (137), fine-tuning for a downstream task is also investigated. Currently, these methods are in development. To be more widely used, we need to further investigate the transferability of the learned models and representations further. In particular, we believe that studies on language models can be explored. Language models are still improved in NLP, with larger models being pursued. The application of these language models in repertoire analysis is also to be investigated.

The other approach is to combine multiple models to exploit more information in repertoire datasets. The hypothesis test-based methods tend to make predictions based on a tiny subset of specific TCRs, especially public TCRs, and ignore most of the other TCRs in the dataset. In other words, these methods are based on the exact match. This is contrary to a certain class of motif-based or deep-learning-based methods that exploit all the sequences in a sample by encoding them with a fixed-length feature vector. In other words, these methods are based on fuzzy matches. Actually, our group compared these two types of methods and revealed that they provide different prediction profiles (43). Two fuzzy-match-based methods yielded similar predictions. This is intriguing because the two methods are based on completely different methods (k-mer encoding on repertoire level + GBDT vs. deep learning-based feature encoding + attention mechanism). On the other hand, a hypothesis-based method yielded very different predictions. This result suggests that these methods may utilize different information and that ensembling these approaches may result in a better performance on smaller datasets.

While the repertoire data may possess the remaining information that can be further exploited, a T cell population cannot be characterized solely by the sequence information of the TCR repertoire. We cannot predict all the nature of TCRs only from the sequence information. Moreover, important information is missing. For example, T-cell subpopulations cannot be determined by sequence data itself. Therefore, the integration of multimodal information is a promising direction for further repertoire analysis. Most of the methods we reviewed in this paper do not employ information other than TCR sequences except one that integrates the physicochemical properties of amino acids to repertoire datasets (90). We may accommodate a lot more sources to analyze the repertoire dataset. Actually, multi-omics analysis is recently explored (181, 182). The multi-omics approach is usually used with single-cell sequencing to connect multiple data at the single-cell level. Currently, such multi-omics data is not yet popularly employed. However, some interesting findings have been reported. For example, single-cell analysis of RNA-seq and CDR3 revealed the correlation between the gene expression and the frequent CDR3 sequences (182). Another source may come from the 3D structure estimation methods, as the nature of a TCR sequence is determined by the binding affinity to antigens. A recent paper (183) encodes a BCR sequence to a feature vector using the estimated 3D structure of the B cell receptor. Another paper (184) utilizes 3D structure information to predict peptides that bind well with a pair of TCR and MHC. In the paper, a binding score matrix between peptide residues and TCR residues is learned from the existing TCR-pMHC structures. The matrix is then used to calculate the possible alternative peptide of the TCR and MHC.

Toward this direction, hand-crafted models, which exploit specific information based on human understanding, can be effectively utilized to complement the data-driven models by DL. By considering the fact that Alphafold2 (116) was realized by the combination of a feature extraction method and loss function based on chemical insights, it would be promising to unite hand-crafted models with data-driven ones and to integrate multimodal data in repertoire analysis.

## Author Contributions

YK, RY: writing of the manuscript, TA, TK: writing of the manuscript and supervision. All authors contributed to the article and approved the submitted version.

## Funding

This research is supported by the JSPS KAKENHI Grant Numbers 19H05799, 19K20408, 20H03441, and by the JST CREST Grant Number JPMJCR2011.

## Conflict of Interest

The authors declare that the research was conducted in the absence of any commercial or financial relationships that could be construed as a potential conflict of interest.

## Publisher’s Note

All claims expressed in this article are solely those of the authors and do not necessarily represent those of their affiliated organizations, or those of the publisher, the editors and the reviewers. Any product that may be evaluated in this article, or claim that may be made by its manufacturer, is not guaranteed or endorsed by the publisher.

## Footnotes

1
http://www.imgt.org/.

2
https://www.ncbi.nlm.nih.gov/sra/.

3
https://www.insdc.org/.

4
https://github.com/ncbi/sra-tools.

5
https://vdjserver.org/.

6
https://clients.adaptivebiotech.com/immuneaccess.

7
https://gateway.ireceptor.org/.

8
http://bioinfo.life.hust.edu.cn/TCRdb/#/.

9
https://github.com/uio-bmi/compairr.

10
https://github.com/GreiffLab/immuneREF.

11
https://immunarch.com/.

12
https://clients.adaptivebiotech.com/pub/covid-2020.
